# Impact of the Number of Positive Pelvic Lymph Nodes on Risk of Para-Aortic Recurrence in Patients with Clinically Early Cervical Cancer Treated by a Radical Hysterectomy and Pelvic Lymphadenectomy

**DOI:** 10.3390/cancers17010023

**Published:** 2024-12-25

**Authors:** Felix J. M. Schoonhoven, Johanna W. M. Aarts, Guus Fons, Lukas J. A. Stalpers, Luc R. C. W. van Lonkhuijzen, Jacobus van der Velden, Constantijne H. Mom

**Affiliations:** 1 Department of Gynecological Oncology, Centre for Gynecologic Oncology Amsterdam (C.G.O.A.), Amsterdam University Medical Center, 1081 HV Amsterdam, The Netherlands; f.j.m.schoonhoven@amsterdamumc.nl (F.J.M.S.); j.w.m.aarts@amsterdamumc.nl (J.W.M.A.); g.fons@amsterdamumc.nl (G.F.); l.r.vanlonkhuijzen@amsterdamumc.nl (L.R.C.W.v.L.); c.mom@amsterdamumc.nl (C.H.M.); 2 Department of Radiation Oncology, Amsterdam University Medical Center, 1055 AZ Amsterdam, The Netherlands; l.stalpers@amsterdamumc.nl

**Keywords:** cervical cancer, multiple positive lymph nodes, para-aortic recurrence

## Abstract

Background: Guidelines recommend the extension of the pelvic radiotherapy volume to the para-aortic region in locally advanced cervical cancer and ≥3 suspicious pelvic lymph nodes (PLN) on imaging. Whether this recommendation is also valid for clinically early stages is uncertain. The objective of this study was to investigate the para-aortic (PAO) lymph node recurrence rate in patients with early-stage cervical cancer, ≥3 metastatic PLN, and negative common iliac nodes after a radical hysterectomy followed by pelvic (chemo)radiotherapy without extension to the PAO region. Methods: Consecutive patients, surgically treated between 2000 and 2020, with FIGO 2009 stage IB2-IIA1 and positive PLN, were included in this retrospective cohort study. The frequency of PAO recurrences, disease-free survival, and overall survival were analyzed in patients with <3 versus ≥3 positive PLN. Results: In 127 patients, the isolated PAO recurrence rate was 2/88 (2.3%) versus 1/39 (2.6%) for patients with <3 versus ≥3 positive PLNs, respectively (*p* = 0.671). The 5-year disease-free survival (87.3% versus 73.7%; *p* = 0.088) and the overall survival (90.7% versus 76.5%; *p* = 0.355) between patients with <3 versus ≥3 positive PLN was not significantly different. Conclusions: Isolated PAO nodal recurrence rate in women with early-stage cervical cancer after radical hysterectomy and pelvic lymphadenectomy, with positive PLN but negative common iliac nodes, followed by pelvic (chemo)radiotherapy, is low and did not differ between the groups with <3 versus ≥3 positive PLN. This makes it unlikely that the inclusion of the PAO region in the adjuvant radiotherapy volume would result in a better oncological outcome.

## Simple Summary

The extension of the radiotherapy field from only the pelvic region to a pelvic and para-aortic (PAO) region in advanced cervical cancer depends on the presence of common iliac and/or PAO lymph nodes suspicious for metastases on a CT scan or PET CT scan. However, in the presence of multiple tumor-positive pelvic lymph nodes (≥3), this extension of the radiotherapy field is also recommended because of the substantial risk of PAO metastases. If this risk of PAO metastases is also substantial in clinically early cervix cancer with multiple positive pelvic lymph nodes, it is controversial. We found a risk of 2.4% for isolated PAO recurrence after a radical hysterectomy and pelvic lymphadenectomy followed by adjuvant pelvic radiotherapy of early cervix cancer, where ≥3 positive pelvic lymph nodes were found. This frequency did not differ from patients with <3 positive pelvic nodes. The fact that the frequency of PAO recurrences is relatively low makes it very unlikely that the extension of the radiotherapy field to the PAO region will result in better survival for these patients with clinical early cervix cancer.

## 1. Introduction

Cervical cancer is one of the most common cancers in women worldwide, with over 600,000 women diagnosed with the disease in 2020 [1]. An important prognostic factor is the presence of pelvic and para-aortic (PAO) lymph node metastases. The incidence of pelvic metastases increases with the International Federation of Gynecology and Obstetrics (FIGO) 2009 stage, ranging from 2% to 36% in clinically early-stage disease (IA/B) to 38–50% in clinically late-stage disease (II-IV). PAO metastases are less common, with a rate of <5% in early-stage disease, increasing to 30–50% in advanced-stage disease [2].

The standard treatment for patients with clinically early-stage disease is a radical hysterectomy and pelvic lymphadenectomy. The advanced-stage disease is treated with concurrent chemotherapy and external beam pelvic radiotherapy combined with brachytherapy [3]. For patients with advanced-stage disease and suspicious PAO and/or common iliac lymph nodes on imaging or proven lymph node metastasis by histology, it is recommended to include the common iliac and PAO regions in the standard pelvic radiotherapy volume [3,4,5]. However, the ESGO guideline also recommends prophylactically including the PAO region in the radiotherapy volume in patients without suspicious nodes in the common iliac and PAO regions but with three or more suspicious pelvic nodes on imaging [3]. Other guidelines either recommend not to extend the standard pelvic radiotherapy volume [6] or do not specifically mention the treatment policy in this context [5]. The EMBRACE II study also recommends prophylactic PAO radiotherapy for patients with three or more suspicious pelvic nodes on imaging [7]. Whether this recommendation for prophylactic PAO radiotherapy in advanced-stage cervical cancer is also justified for clinically early-stage disease with multiple pathologically confirmed positive pelvic nodes is debatable.

In a large retrospective study of patients with FIGO 2009 stage IB, IIA, or IIB cervical cancer, the variable “multiple positive pelvic nodes” was an independent predictor of PAO recurrence after radical hysterectomy [8]. However, a randomized trial of adjuvant extended field radiotherapy in surgically treated early cervical cancer patients with multiple positive pelvic nodes did not show a survival benefit when including the PAO region [9]. Therefore, a critical analysis of the risk of PAO metastasis or recurrence in patients with three or more positive pelvic nodes in clinically early-stage cervical cancer is needed. The primary objective of this study was to evaluate the likelihood of isolated para-aortic lymph node recurrence in patients with clinically early-stage cervical cancer with three or more versus less than three pathologically confirmed positive pelvic nodes after radical hysterectomy and pelvic lymphadenectomy followed by pelvic (chemo)radiotherapy. The secondary objective was to compare the disease-free survival (DFS), disease-specific survival (DSS), and overall survival (OS) between the two groups.

## 2. Materials and Methods

In this retrospective cohort study, data were collected from the database of the Department of Gynecological Oncology at the Amsterdam University Medical Center in the Netherlands. All consecutive patients with clinically early-stage cervical cancer treated with a radical hysterectomy and pelvic lymphadenectomy between January 2000 and December 2020 and with positive pelvic nodes were included. The staging was performed according to the guidelines of the FIGO 2009 staging system. Pathology specimens were analyzed prospectively by a gynecological pathologist and discussed in a multidisciplinary tumor board. Patients with squamous cell carcinoma, adenosquamous carcinoma, or adenocarcinoma were included. All patients were treated with an open, type C2, radical hysterectomy and pelvic lymph node dissection [10]. A modified type I pelvic lymph node dissection was performed [11]. The modification was in the area of the obturator fossa, where lymph nodes were removed both above and below the obturator nerve. Patients with high-risk factors, i.e., positive pelvic nodes, parametrial invasion, or irradical resection, were treated with adjuvant pelvic radiotherapy and concurrent chemotherapy. The parametrial invasion was defined as the direct extension of the tumor into the parametrium and/or the presence of lymphovascular space invasion (LVSI) in the parametrium. Irradical resection was defined as a tumor < 1 mm from the dissection margin. Standard pelvic radiotherapy consisted of 45 Gy in 25 fractions of 1.8 Gy using either 3D conformal and gradually IMRT or arc-rotation radiotherapy. Patients with positive vaginal margins received additional brachytherapy to the vaginal vault. Chemotherapy consisted of weekly cisplatin 40 mg/m^2^ for a total of 6 cycles. In patients with squamous cell cancer and only one high-risk factor, chemotherapy was omitted. For patients with positive common iliac and/or para-aortic lymph nodes, the radiation volume was increased to also include the para-aortic region. These patients were excluded from further analysis. The patients included in the study were divided into two groups. The first group consisted of patients with less than three positive PLNs, and the second group of patients with three or more positive PLNs. The following data were retrieved from patient records and pathology reports: age, clinical tumor diameter, FIGO stage, invasion depth, positive margins, histological type, LVSI, parametrial involvement, number and location of positive nodes, and type of adjuvant treatment (chemoradiotherapy or radiotherapy).

Patients were seen for follow-up every 3 months during the first year, every 6 months during the second and third years, and once a year thereafter. Follow-up consisted of a medical history and physical examination. If a recurrence was clinically suspected, diagnostic tests such as MRI, CT scan, and/or PET-CT scan, serum tumor marker(s), and/or biopsies were performed. Recurrences were stratified as “loco-regional”, defined as a recurrence in the pelvic cavity, and “distant”, defined as a recurrence outside the pelvis. Combined recurrences were defined as a combination of loco-regional and distant recurrence. PAO recurrences were recorded separately either as “combined PAO”, defined as a pelvic recurrence combined with a PAO recurrence component, or “isolated PAO recurrence”, defined as a recurrence in the PAO region with or without distant lymph node recurrence more cranially from the PAO recurrence.

Institutional review board approval was not obtained, as this is not required under Dutch law when anonymized patient data are used in accordance with rules of good clinical practice.

### Statistical Methods

IBM SPSS Statistics (version 28) for Windows was used for analysis. The outcomes were as follows:

- isolated PAO recurrence rate
- PAO recurrence-free survival is defined as the time between the date of the radical hysterectomy and the first diagnosis of the isolated PAO recurrence.
- disease-free survival, defined as the interval between the date of radical hysterectomy and the date of recurrence or the last follow-up visit;
- disease-specific survival, defined as the survival time between the date of radical hysterectomy and death from recurrent cervical cancer or the date of last follow-up;
- overall survival, defined as the survival time between the date of radical hysterectomy and death (from any cause) or the date of the last follow-up visit.

Patients and disease characteristics were described using standard descriptive statistics: median/range for continuous data and frequencies/percentages for categorical data. For continuous data, a Student’s *t*-test was used to compare the groups. For the categorical data, a chi-square analysis or Fischer exact test was used, depending on the expected number of observations. A univariate Kaplan–Meier curve was then plotted for PAO recurrence, overall survival, disease-specific survival, and disease-free survival. For all statistical tests, a two-tailed *p*-value of <0.05 was considered significant.

## 3. Results

**Figure 1:**
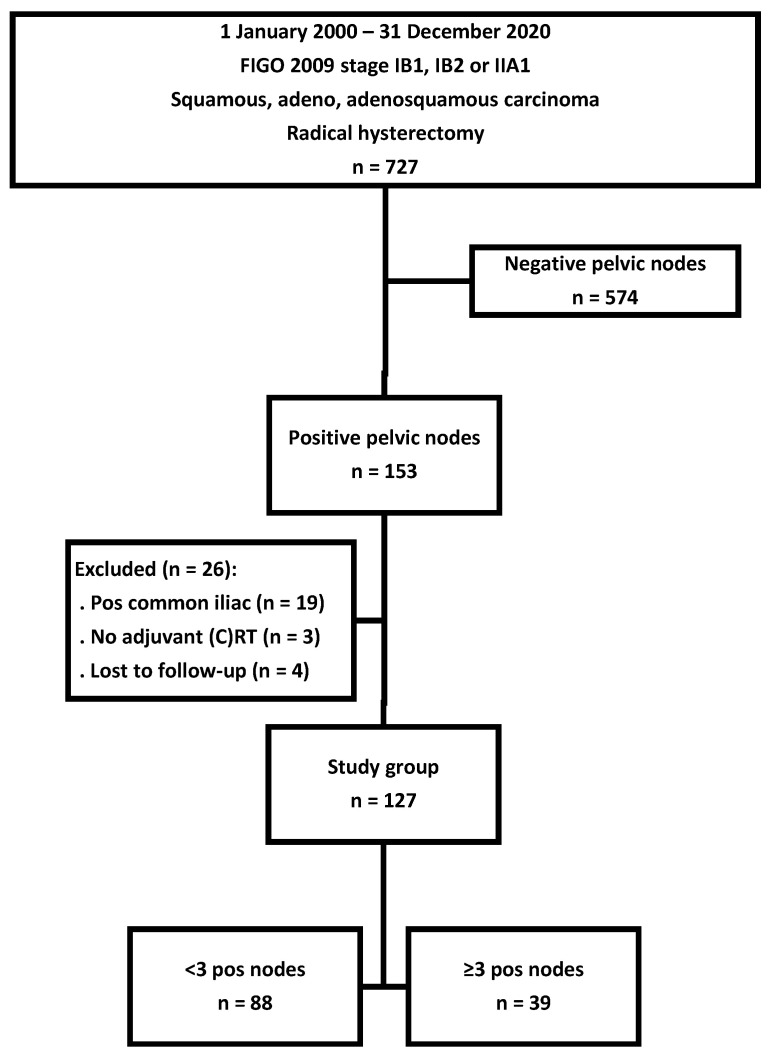
Selection of patients with positive pelvic nodes after radical hysterectomy for stage FIGO 2009 stage IB or IIA1 cervical cancer. Abbreviations: FIGO, International Federation of Gynecology and Obstetrics; pos, positive; (C)RT, (chemo)radiotherapy.

A total of 727 consecutive patients with stage IB or IIA cervical cancer were treated with a radical hysterectomy and pelvic lymphadenectomy. Patients with negative pelvic nodes (n = 574) were excluded. Another 26 patients were excluded for the following reasons: the presence of positive common iliac nodes (n = 19), no adjuvant therapy administered (n = 3), and the loss to follow-up (n = 4). The remaining 127 patients formed the basis of this study (Figure 1).

The median and mean number of removed pelvic lymph nodes were 28 (range 8–52) and 29.1 ± 9.7, respectively. The median and mean number of removed right common iliac nodes were 2 (range 0–8) and 1.6 ± 0.9, respectively, and on the left side, 1 (range 0–6) and 0.9 ± 1.0, respectively. Of the 127 patients, 88 patients had <3 positive nodes, and 39 patients had ≥3 positive nodes.

In Table 1, the clinical and pathological characteristics of the patients are summarized. In patients with <3 positive PLN, the median tumor diameter was smaller (30 mm vs. 37 mm, *p* = 0.044), fewer positive margins were found (3.4% vs. 28.2%, *p* < 0.001), and less parametrial invasion was present (21.6% vs. 46.2%, *p* = 0.003) compared with patients with ≥3 positive PLN. Patients with ≥3 positive PLN had more adjuvant concurrent chemotherapy and radiotherapy compared to patients with <3 positive PLN (89.7% vs. 55.7%, *p* < 0.001).

After a median follow-up of 75 months (range 30–271) for censored patients, the crude recurrence rate was 13/88 (14.8%) in the <3 positive PLN group versus 10/39 (25.6%) in the ≥3 positive PLN group (*p* = 0.113). 

The frequency of isolated PAO recurrences did not differ between the groups with <3 (2/88) versus ≥3 (1/39) positive PLN (2.3% vs. 2.6%, *p* = 0.671). All three patients with an isolated PAO recurrence also had left supraclavicular nodal metastases. The diagnosis was confirmed by a PET CT scan, showing suspicious PET-positive nodes both in the lower PAO and left supraclavicular area and by histopathology of the supraclavicular nodes in all three patients. The one patient with ≥3 positive pelvic nodes and an isolated PAO recurrence had one negative common iliac node on each side.

When the total frequency of PAO recurrences (isolated and combined) was considered, there was no difference in the frequency of PAO recurrence between patients with <3 vs. ≥3 positive PLN (5/88 (5.7%) vs. 3/39 (7.7%), *p* = 0.503). The location of recurrences in the two groups is shown in Table 2. There was no significant difference in the location of recurrences between the two groups.

**Figure 2:**
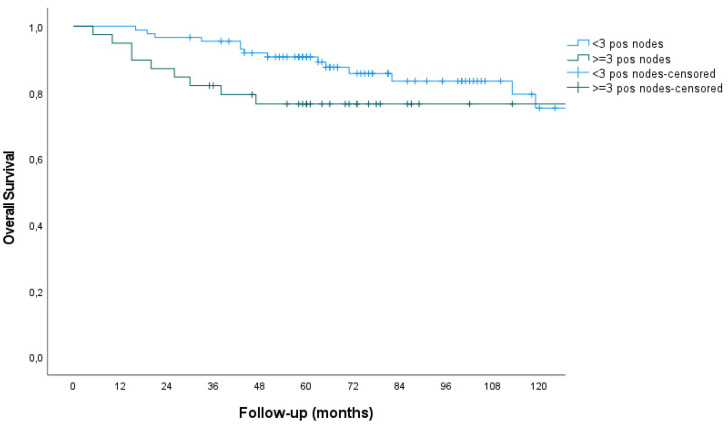
Kaplan–Meier curves of overall survival rates for patients with <3 or ≥3 positive pelvic lymph nodes (5-year OS: 90.7% versus 76.5%, respectively; *p* = 0.355).

The 5-year overall survival was not different between patients with <3 versus ≥3 positive pelvic nodes (90.7% versus 76.5%, respectively, *p* = 0.355). (Figure 2). The mean overall survival was 183 months versus 213 months, respectively. The 5-year disease-specific survival and disease-free survival rates were not different either (91.8% versus 83.9%; *p* = 0.472 and 87.3% versus 73.7%; *p* = 0.088, respectively), nor were the isolated PAO recurrence-free survival rates between the two groups (98.8% versus 96.9%, *p* = 0.774). The mean disease-free survival was 196 months versus 205 months for patients with <3 versus ≥3 positive pelvic nodes, respectively.

## 4. Discussion

In this retrospective cohort study, we found a low frequency of isolated PAO recurrences (2.6%) in patients with ≥3 positive pelvic nodes after radical hysterectomy with adjuvant (chemo)radiotherapy restricted to the pelvic area for clinically early-stage cervical cancer and negative common iliac nodes. This low frequency did not differ from the frequency of isolated PAO recurrences in patients with <3 positive nodes (2.3%). This low frequency of PAO recurrences makes it very unlikely that prophylactic extended field radiotherapy will improve the oncological outcome in patients with ≥3 positive pelvic nodes.

We found no impact of the number of positive pelvic nodes on PAO recurrence risk. This contrasts with a large retrospective cohort study by Matsuo et al. [8] consisting of 4663 patients with stage IB to IIB, all treated with a type III radical hysterectomy and pelvic lymph node dissection, non-suspicious PAO lymph nodes on pre-operative imaging and during surgery. The number of positive lymph nodes was an independent predictor of the risk of PAO recurrence. Patients with multiple (>1) pelvic node metastases showed a 19% risk of PAO recurrence. We, in contrast, found risks of 7.3% combined loco-regional and PAO recurrence and 2.4% isolated PAO recurrence in patients with ≥3 positive pelvic nodes. This discrepancy can probably be explained by differences in clinical and pathological characteristics, i.e., by more favorable patients in our cohort. The study of Matsuo et al. included patients with clinical stage IIB, whereas we included patients with clinical stages IB to IIA. More importantly, in the Matsuo study, information on the status of the common iliac nodes was not available, resulting in the inclusion of unfavorable patients with positive common iliac nodes and, therefore, more PAO recurrences. We excluded the patients with positive common iliac nodes because, based on many international guidelines, these patients are always treated with prophylactic PAO radiotherapy, irrespective of the number of positive pelvic nodes [3,4,5].

In a study of 112 patients with clinical stage IB to IIB, all treated by a radical hysterectomy and pelvic as well as PAO lymph node dissection, the presence of multiple (i.e., >1) positive pelvic lymph nodes was an unfavorable risk factor for PAO metastases [12]. A 52.1% incidence of PAO metastases in patients with >1 positive pelvic node (and 3.1% in patients with 0–1 positive pelvic node) was found. This high risk of PAO metastases is likely caused by selection bias because the 112 patients with both pelvic and PAO lymph node dissection had been selected from a larger cohort of 428 patients, of whom the other 316 patients only had a pelvic node dissection. The selection criteria were not mentioned [12]. A recent study by Holloway et al. (2023) on the risk of PAO recurrences in clinically early-stage (IA and IB) cervical cancer after radical hysterectomy showed a 0% risk in patients who had no PAO dissection [13]. However, in that study, only 28/265 (10.6%) patients had positive pelvic nodes, and information on the number of positive nodes was not reported. Therefore, this study does not answer the question of the risk of PAO metastasis/recurrences in patients with clinical stage IB and multiple positive nodes.

The fact that we did not find a relationship between the presence of multiple positive pelvic nodes and the risk of PAO recurrence, while the aforementioned studies did, is most likely caused by the fact that our cohort only consisted of patients with clinically early cervical cancer and negative common iliac nodes.

We did not find a significant difference in survival between patients with <3 versus ≥3 positive pelvic nodes (91.9% versus 75.2%, respectively; *p* = 0.136). This contrasts with other studies showing a number of positive pelvic nodes to be an independent risk factor for recurrence and survival [14,15]. The relatively low numbers in our study could have been the cause of this lack of impact on survival. On the other hand, the referenced studies do not mention the inclusion or exclusion of positive common iliac nodes in the studied patient population. Moreover, in contrast to our study, in the study by Bogani et al. [15], patients with clinical stage IIA2 and IIB and patients with positive PAO nodes were included, resulting in a group of patients with additional poor prognostic factors, which may have had an impact on their survival analyses.

The implication of our finding that we, in contrast to others, did not find a higher risk of PAO recurrence in patients with multiple positive pelvic nodes would be that extension of the radiotherapy field to the PAO region is not recommended for this specific subgroup of patients. In the context of the conflicting results, it is interesting to see that a randomized controlled trial found no improved survival or disease-free survival benefit from EFRT in patients with FIGO 2009 stage IB to II, with two or more positive pelvic nodes and negative common iliac nodes [9]. However, this trial was underpowered, with only 126 patients randomized. It is clear that more data are needed to analyze the risk of PAO metastasis or recurrence for the specific group of patients with clinically early cervical cancer (FIGO 2009 stage IB) treated by surgery with multiple positive pelvic nodes and negative common iliac nodes before firm conclusions can be drawn about the impact of prophylactic EFRT.

The limitations of our study include the analysis of retrospective data over a twenty-year time period in a relatively small population with only 127 patients. The introduction of more advanced imaging techniques, such as PET/CT, over the last decade could have had an impact on the selection of patients for primary surgery. Furthermore, a causal relationship between the number of PLN and PAO recurrences cannot be established. Future research should provide a better answer using multivariate analysis in a larger dataset. This could provide a better understanding of which confounders contribute the most to the PAO recurrence. If these confounders are found, a better assessment of the need for EFRT can be made. The strength of the study is the homogeneous data set of consecutive patients who were all treated following the same protocol, both surgically and with radiotherapy. While many other studies on this topic either included patients with stage IIB and/or did not differentiate between patients with or without common iliac metastases, we only included patients with clinical stages IB to IIA with positive pelvic nodes and negative common iliac nodes.

## 5. Conclusions

This study shows that the risk of isolated PAO recurrences is low in a selected group of patients with clinically early cervical cancer treated by a radical hysterectomy and pelvic lymphadenectomy with ≥3 positive pelvic nodes and negative common iliac nodes without adjuvant EFRT. Based on these data and supported by the results of one randomized controlled trial, there seems to be no strong indication to include the paraaortic volume in the prophylactic adjuvant radiotherapy in this particular group of patients.

## Figures and Tables

**Table 1:** Patient and tumor characteristics of patients with early cervical cancer and three or more versus less than three positive PLN.

Variable	<3 Pos Nodes (n = 88)	≥3 Pos Nodes (n = 39)	*p*-Value
**Median age (range)**	**42 (23–71)**	**41 (28–70)**	0.523
Median tumor diameter (mm, range)	30 (8–80)	37 (15–80)	**0.044**
FIGO (2009)			
IB1 (n = 83)	61/88 (69.3%)	22/39 (56.4%)	
IB2 (n = 27)	17/88 (19.3%)	10/39 (25.6%)	
IIA1 (n = 17) *	10/88 (11.4%)	7/39 (18.0%)	0.355
Histological type			
Squamous (n = 87)	59/88 (67.0%)	28/39 (71.8%)	
Non-squamous (n = 40)	29/88 (33.0%)	11/39 (28.2%)	0.376
LVSI			
Negative (n = 40)	30/88 (34.1%)	10/39 (25.6%)	
Positive (n = 87)	58/88 (65.9%)	29/39 (74.4%)	0.232
Parametrial invasion			
Absent (n = 90)	69/88 (78.4%)	21/39 (53.8%)	
Present (n = 37) ^†^	19/88 (21.6%)	18/39 (46.2%)	**0.003**
Positive surgical margin			
Absent (n = 113)	85/88 (96.6%)	28/39 (71.8%)	
Present (n = 14) ^‡^	3/88 (3.4%)	11/39 (28.2%)	**<0.001**
Adjuvant therapy			
Radiotherapy (n = 43)	39/88 (44.3%)	4/39 (10.3%)	
Chemoradiotherapy (n = 84)	49/88 (55.7%)	35/39 (89.7%)	**<0.001**

* FIGO stage IIA with a diameter ≤ 4 cm diagnosed before 2009 was converted to FIGO 2009 stage IIA1. ^†^ Parametrial invasion on the basis of direct invasion (n = 23); LVSI in parametrium (n = 17). ^‡^ Positive surgical margin on the basis of close margin (n = 5); positive vaginal margin (n = 5); positive dorsal or ventral anatomical margin (n = 4); positive parametrial margin (n = 1). Abbreviations: pos, positive; mm, millimeter; FIGO, International Federation of Gynecology and Obstetrics.

**Table 2:** Distribution of recurrence location in patients with <3 versus ≥3 positive PLN.

Recurrence Location	<3 Pos PLN	≥3 Pos PLN	*p*-Value
**Isolated pelvic**	**2/88 (2.3%)**	**2/39 (5.1%)**	0.354
Pelvic and distant (ex PAO)	1/88 (1.1%)	2/39 (5.1%)	0.141
Isolated distant (ex PAO)	3/88 (3.4%)	3/39 (7.7%)	0.230
Pelvic and PAO combined	3/88 (3.4%)	2/39 (5.1%)	0.533
Isolated PAO	2/88 (2.3%)	1/39 (2.6%)	0.774
Unknown	2/88 (2.3%)	0/39 (0.0%)	0.412

Abbreviations: ex, excluding; PAO, para-aortic; pos, positive; PLN, pelvic lymph nodes.

## Data Availability

The data presented in this study are available on request from the corresponding author.
